# Vitiligo secondary to immunosuppressants: a pharmacovigilance study of the FDA Adverse Event Reporting System

**DOI:** 10.3389/fimmu.2026.1707427

**Published:** 2026-03-12

**Authors:** Ying Jia, Hanzhang Xie, Yixuan Yang, Bingnan Cui, Junhui Wang, Zhanshuo Xiao

**Affiliations:** 1 Department of Dermatology, Guang’anmen Hospital, China Academy of Chinese Medical Sciences, Beijing, China; 2 Beijing University of Chinese Medicine, Beijing, China

**Keywords:** adverse event, drug-induced vitiligo, FAERS, pharmacovigilance study, vitiligo

## Abstract

**Background:**

Vitiligo is an acquired depigmentation disorder affecting individuals worldwide. The potential link between immunosuppressants and the onset or exacerbation of vitiligo remains a topic of clinical concern.

**Objective:**

This study aims to evaluate the association between various immunosuppressants and vitiligo using data from the FDA Adverse Event Reporting System (FAERS).

**Methods:**

A retrospective pharmacovigilance analysis was conducted using FAERS data from January 2004 to June 2024. Vitiligo cases were identified through the Medical Dictionary for Regulatory Activities (MedDRA) terminology. Disproportionality analysis was performed using the Reporting Odds Ratio (ROR), Proportional Reporting Ratio (PRR), Bayesian Confidence Propagation Neural Network (BCPNN), and Empirical Bayes Geometric Mean (EBGM) to detect significant drug-event associations.

**Results:**

A total of 435 vitiligo-related adverse event (AE) reports were identified. The United States, Canada, France, Germany, and Brazil contributed the most reports. Vitiligo reports were more frequent in female patients, particularly within the 18–65 age group. The primary indications for immunosuppressant use included psoriasis, rheumatoid arthritis, psoriatic arthropathy, Crohn’s disease, and ulcerative colitis. Adalimumab (74 cases) and secukinumab (52 cases) accounted for the highest number of reports. Significant signals were detected for alemtuzumab, ixekizumab, ustekinumab, secukinumab, guselkumab, and risankizumab.

**Conclusion:**

This study highlights the importance of continuous pharmacovigilance in monitoring potential adverse events associated with immunosuppressants. The observed association between specific immunosuppressants and vitiligo suggests a need for further research to elucidate underlying mechanisms and develop strategies to mitigate these potential AEs.

## 1 Introduction

Vitiligo is a common acquired pigmentary disorder characterized by the development of white macules due to the loss of functional melanocytes (1). The disease has an estimated global prevalence of 0.5–2% of the population worldwide (2). While the exact pathogenesis of vitiligo remains incompletely understood, it is widely believed that autoimmune, genetic, and environmental factors contribute to its development (3). Immunosuppressants play a significant role in the treatment of skin-related diseases by modulating immune responses to control symptoms and suppress excessive immune activity, particularly in the management of inflammatory skin conditions such as psoriasis (4) and autoimmune bullous diseases (5). However, their use is not without risks, and concerns have been raised regarding the potential for immunosuppressants to trigger or exacerbate other skin conditions, including vitiligo (6).

A recent comprehensive pharmacovigilance analysis identified 46 drugs associated with vitiligo signals, significantly broadening the known causative profile (7). Distinct safety signals have been established for a diverse spectrum of immunomodulators, ranging from immune checkpoint inhibitors (targeting PD-1/PD-L1 and CTLA-4) and CDK4/6 inhibitors (8) to novel biologics targeting specific cytokine axes, such as type 2 immune inhibitors (9). Notably, the clinical characteristics and associated risk factors of vitiligo induced by these antineoplastic agents have already been extensively profiled across diverse cancer populations (10). While recent pharmacovigilance studies have broadened the known causative profile of drug-induced vitiligo, they have predominantly focused on immune checkpoint inhibitors or broad classes of immunomodulators. There remains a notable gap in the literature regarding the granular safety profiles of modern biologics, specifically those targeting the IL-17 signaling pathway and the IL-23/Th17 axis. Furthermore, a comprehensive comparative analysis of these novel agents alongside established triggers like alemtuzumab has not been sufficiently explored. Leveraging the FAERS database—a critical resource for monitoring the safety of marketed drugs—this study aims to systematically evaluate and compare the association of these specific biologic agents with vitiligo using robust disproportionality algorithms.

## 2 Methods

### 2.1 Data sources

This retrospective pharmacovigilance study utilized data from the FAERS database, covering the period from January 2004 to June 2024. FAERS is a global repository of AE reports voluntarily submitted by healthcare professionals, consumers, and manufacturers. The database includes detailed information on drugs—such as names, active ingredients, routes of administration, and roles in reported AEs—coded using the Medical Dictionary for Regulatory Activities (MedDRA). Additionally, FAERS classifies drug involvement in AEs with codes indicating primary suspect (PS), secondary suspect, interacting, or concomitant medications. Each report identifies a primary suspect drug, lists one or more AEs, and may include information on other medications the patient was taking. As FAERS relies on spontaneous reporting, it is subject to inherent biases, including under-reporting, regional disparities, and variable reporting rates across healthcare systems.

### 2.2 Study design

The study encompassed FAERS reports from January 2004 to June 2024. Duplicate reports were identified and excluded based on case numbers, with only the most recent version retained for analysis. A case-control analysis was performed using FAERS to investigate the association between drug exposure and reports of vitiligo. The analysis was based on a 2×2 contingency table (**Supplementary Table S1**) comparing the specific immunosuppressant-vitiligo pair against all other drug-event combinations in the full database. “Cases” (cell a) were defined as reports involving the target drug and the target AE (vitiligo). “Controls” for the background comparison (cells c and d) comprised all other reports in the entire FAERS database involving any other drugs (including non-immunosuppressants) and any other adverse events. We utilized the full FAERS database as the reference background, ensuring that the generated signals reflect reporting frequencies relative to the overall drug safety profile.

### 2.3 Data exposure and AEs definition

For the purpose of this study, the MedDRA term for vitiligo was used to identify relevant AEs in the FAERS database. The assessment of drug exposure focused on drugs designated with the ‘primary suspect’ role code. DrugBank was utilized to standardize different drug names, including brand names, generic names, synonyms, or abbreviations, ensuring that all drugs were represented in their standardized generic name format. Each report identified a primary suspect drug and enumerated one or more AEs, which were then classified according to their MedDRA terms.

### 2.4 Statistical analysis

Disproportionality analysis was conducted to identify potential safety signals. In this study, we applied four statistical methods using 2×2 contingency tables: the Reporting Odds Ratio (ROR) and Proportional Reporting Ratio (PRR) as frequentist metrics, and the Bayesian Confidence Propagation Neural Network (BCPNN) and Empirical Bayes Geometric Mean (EBGM) as Bayesian methodologies. These algorithms were used to calculate the association between drugs and vitiligo. The Bayesian methods offer a significant advantage by mitigating the instability of false positives associated with sparse AE reporting. **Supplementary Table S1** provides the detailed formulas, calculation methods, and specific thresholds used for each of these four methods, further clarifying their operational details. To ensure the robustness of the identified signals, strict thresholds were applied. A signal was considered significant only if it met all four of the following criteria simultaneously: (1) the number of reports (N) ≥ 3; (2) the lower limit of the 95% confidence interval of ROR (ROR025) > 1; (3) the PRR ≥ 2 with χ² ≥ 4; and (4) the lower limit of the 95% confidence interval for EBGM (EBGM05) > 2. Additionally, the Information Component lower limit (IC025) > 0 was used as a confirmatory metric. All data processing and statistical analyses were performed using R software, version 4.4.1.

## 3 Results

### 3.1 Case characteristics

**Figure 1:**
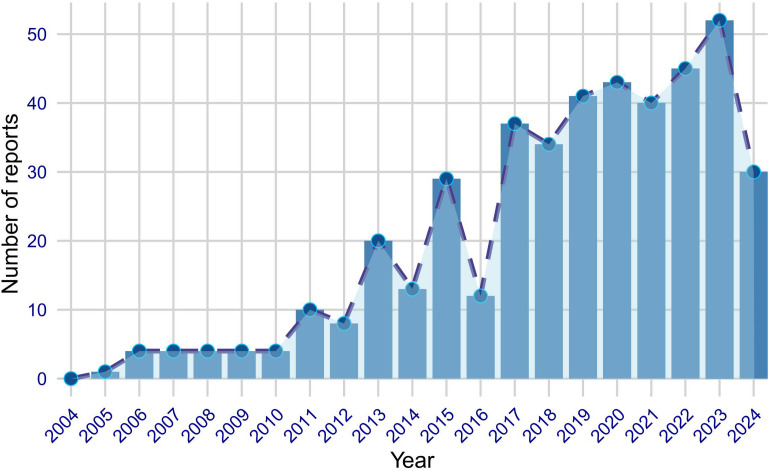
Annual trends in FAERS reports of vitiligo associated with immunosuppressants from January 2004 to June 2024.

Between the first quarter of 2004 and the second quarter of 2024, our study extracted a total of 1726 AE reports of vitiligo associated with immunosuppressants from the database. After removing duplicates, we obtained 435 distinct AE reports related to vitiligo. **Figure 1** illustrates the annual submission of AE reports, showing a notable increase in reports over the years, particularly in recent years. In the analyzed reports, there was a slight female predominance (52.2%) compared to males (32.0%), with a significant number of reports having missing sex data (15.9%). In terms of weight, a majority of the reports had missing weight data (82.8%), with only a small proportion of patients weighing less than 50 kg (0.5%) or more than 100 kg (1.8%), and the rest falling within the 50–100 kg range (14.9%). Regarding age, the majority of reports had missing age data (48.0%), with the reported ages distributed as follows: less than 18 years (3.9%), between 18 and 65 years (40.7%), and between 66 and 85 years (7.1%). The majority of reports were submitted by physicians (36.1%), followed by health professionals (14.0%), other health professionals (12.9%), pharmacists (3.0%), and consumers (32.9%), with a small number of reports missing this information (1.1%). Geographically, the United States accounted for the highest proportion of reports (60.5%), followed by Canada (6.9%), France (4.8%), Germany (3.4%), and Brazil (3.2%). Regarding outcomes, the most frequently reported indications were psoriasis (26.9%), rheumatoid arthritis (9.9%), psoriatic arthropathy (8.5%), crohn’s disease (6.9%), and colitis ulcerative (4.8%). **Table 1** provides a comprehensive overview of the clinical characteristics of the association between immunosuppressants and vitiligo.

**Table 1:** Clinical characteristics of reports on the association between immunosuppressants and vitiligo from January 2004 to June 2024.

Characteristics	Case number	Case proportion
**Number of events**	435	
Sex		
F	227	52.2%
M	139	32.0%
Missing	69	15.9%
Weight(kg)		
<50	2	0.5%
>100	8	1.8%
50∼100	65	14.9%
Missing	360	82.8%
Age(year)		
<18	17	3.9%
>85	1	0.2%
18-65	177	40.7%
66-85	31	7.1%
Missing	209	48.0%
Reported Person		
Consumer	143	32.9%
Health Professional	61	14.0%
Physician	157	36.1%
Other health-professional	56	12.9%
Pharmacist	13	3.0%
Missing	5	1.1%
Reported Countries (top five)		
United States	263	60.5%
Canada	30	6.9%
France	21	4.8%
Germany	15	3.4%
Brazil	14	3.2%
Indications (top five)		
Psoriasis	117	26.9%
Rheumatoid arthritis	43	9.9%
Psoriatic arthropathy	37	8.5%
Crohn’s disease	30	6.9%
Colitis ulcerative	21	4.8%

### 3.2 Immunosuppressants used for vitiligo

Among the 1726 AE reports of vitiligo associated with immunosuppressants identified in this study, a total of 491 unique drug names were listed. After merging different names, including brand and generic names, we arrived at 25 distinct medications which have at least 3 reports. Adalimumab topped the list with 74 reports, followed by secukinumab (n=52), infliximab (n=46), and etanercept (n=38) (**Table 2**). We performed a disproportionality analysis on the 25 medications with more than three reports and initially identified 6 immunosuppressants that met the criteria of the ROR algorithm. To more accurately elucidate the relationship between medications and AEs of vitiligo, we further analyzed immunosuppressants that satisfied all four disproportionality analysis methods (**Table 3**). Among the 6 immunosuppressants with significant associations, we found a variety of immunosuppressant classes, including monoclonal antibodies and interleukin inhibitors. Notably, alemtuzumab had the highest ROR of 9.37, indicating a strong statistical association with reports of vitiligo. Ixekizumab demonstrated a very high ROR of 8.82, suggesting a potentially strong signal for vitiligo. Ustekinumab and guselkumab had elevated RORs of 6.03 and 5.63, respectively, indicating a significant link to AEs. Although tofacitinib had a relatively high number of reports (n=34), it did not meet the strict criteria of all four disproportionality analyses. This distinction is crucial given its pharmacological context.

**Table 2:** Immunosuppressants associated with vitiligo (signals identified using the ROR method, with drugs highlighted in bold).

Drug	ATC code	Case numbers	ROR
Adalimumab	L04AB04	74	1.23
**Secukinumab**	L04AC10	52	**3.83**
**Infliximab**	L04AB02	46	**1.76**
Etanercept	L04AB01	38	0.89
**Tofacitinib**	L04AF01	34	**2.63**
**Ustekinumab**	L04AC05	30	**6.03**
**Alemtuzumab**	L04AG06	20	**9.37**
Lenalidomide	L04AX04	17	0.75
**Risankizumab**	L04AC18	16	**4.39**
**Apremilast**	L04AA32	15	**1.88**
**Ixekizumab**	L04AC13	15	**8.82**
**Guselkumab**	L04AC16	8	**5.63**
Tocilizumab	L04AC07	8	0.95
Vedolizumab	L04AG05	8	1.01
Tacrolimus	L04AD02	7	1.21
Teriflunomide	L04AK02	7	1.56
Certolizumab pegol	L04AB05	6	0.92
Abatacept	L04AA24	5	0.62
Dimethyl Fumarate	L04AX07	5	0.53
Fingolimod	L04AE01	5	0.57
Ocrelizumab	L04AG08	5	0.82
Belimumab	L04AG04	4	1.67
Cyclosporine	L04AD01	4	0.89
Golimumab	L04AB06	3	0.87
Upadacitinib	L04AF03	3	0.71

**Table 3:** Drugs significantly associated with vitiligo due to immunosuppressants (consistent with all four disproportionality analysis methods).

Drug	ATCcode	CaseNumbers	ROR (95%Cl)	PRR (χ^2^)	EBGM (EBGM05)	IC (IC025)
Alemtuzumab	L04AG06	20	9.37 (6.03-14.57)	9.37 (147.84)	9.27 (6.41)	3.21 (1.55)
Ixekizumab	L04AC13	15	8.82 (5.3-14.66)	8.82 (103.04)	8.75 (5.72)	3.13 (1.46)
Ustekinumab	L04AC05	30	6.03 (4.2-8.65)	6.03 (123.73)	5.94 (4.39)	2.57 (0.9)
Guselkumab	L04AC16	8	5.63 (2.81-11.27)	5.63 (30.3)	5.61 (3.13)	2.49 (0.82)
Risankizumab	L04AC18	16	4.39 (2.68-7.18)	4.39 (41.44)	4.35 (2.88)	2.12 (0.46)
Secukinumab	L04AC10	52	3.83 (2.9-5.04)	3.83 (105.26)	3.74 (2.97)	1.9 (0.24)

Signals were defined as significant if all four criteria were met: ROR 95% CI >1, PRR χ² >4, EBGM05 >2, and IC025 >0.

## 4 Discussion

Our study presents compelling evidence of a significant association between certain immunosuppressants and the onset of vitiligo. These medications, including secukinumab, alemtuzumab, ixekizumab, ustekinumab, guselkumab, and risankizumab, are primarily used to treat autoimmune conditions like psoriasis and rheumatoid arthritis. Among the immunosuppressants reported in the FAERS database, the seven most frequently implicated agents were adalimumab (n=74), secukinumab (n=52), infliximab (n=46), etanercept (n=38), tofacitinib (n=34), ustekinumab (n=30), and alemtuzumab (n=20). Except for etanercept and adalimumab, all demonstrated high Reporting Odds Ratios (RORs), indicating a statistically significant association with the development of vitiligo. However, tofacitinib requires distinct interpretation. Unlike the other agents, it did not meet the simultaneous criteria of all four disproportionality analyses. Given its therapeutic application in vitiligo (11, 12), these reports likely reflect confounding by indication or treatment failure rather than a true drug-induced adverse event. Notably, alemtuzumab, ixekizumab, and ustekinumab exhibited the highest ROR and Proportional Reporting Ratio (PRR) values—9.37, 8.82, and 6.03, respectively—suggesting a strong potential safety signal for vitiligo in certain patient populations. Furthermore, the EBGM and Information Component (IC) values confirm the consistency and biological plausibility of these associations. Alemtuzumab, for instance, has an EBGM value of 9.27 and an IC value of 3.21, reinforcing the statistical strength of the observed association. Ruck T et al. has reported 3 patients with multiple sclerosis showing vitiligo after treatment with alemtuzumab (13). In addition, a tertiary care hospital in Spain documented that 2 out of 133 patients treated with alemtuzumab developed vitiligo (14).

Secukinumab, an anti-interleukin-17A monoclonal antibody, has demonstrated efficacy in controlling symptoms of ankylosing spondylitis (15) and psoriasis (16). However, growing evidence has linked its use to the development of vitiligo, with several cases reported in the literature (17–21) and 52 additional cases identified in the FAERS database. Similarly, ixekizumab, which specifically targets IL-17A and shows strong efficacy in the treatment of psoriasis, has likewise been linked to multiple reported cases of vitiligo (22–27). Therefore, clinicians should remain cautious about the possibility of AEs, such as vitiligo, when prescribing immunosuppressants for psoriasis, ankylosing spondylitis and other conditions. Even drugs with a relatively low number of reports, such as risankizumab, ustekinumab (28) and guselkumab (29), demonstrate high ROR and PRR values, highlighting their association with vitiligo reports. This underscores the importance of vigilant monitoring for potential risk factors associated with these drugs, particularly in patient populations predisposed to developing vitiligo. These findings have potential implications for both drug development and safety regulation, highlighting the need for continued investigation into the underlying mechanisms by which certain drugs may be associated with vitiligo, as well as the development of preventive or mitigating strategies. Moreover, our study underscores the importance of comprehensive AE monitoring across all phases of drug development, including clinical trials, rather than limiting surveillance to the post-marketing stage, in order to detect and address potential safety concerns at an earlier stage.

Vitiligo is a complex autoimmune disorder characterized by the appearance of depigmented patches on the skin. It involves a multifaceted etiology with genetic, environmental, and stochastic factors (30). While its molecular pathogenesis is primarily driven by CD8+ T cell-mediated destruction of melanocytes via the interferon-gamma (IFN-γ) signaling axis (31, 32), emerging evidence suggests that cytokines such as interleukin-17 (IL-17) and interleukin-23 (IL-23) also play a significant role in the pathogenesis of vitiligo (33). Elevated levels of these cytokines have been detected in both the serum and skin lesions of affected individuals (34). However, treatments which target these cytokines have not been shown to reverse vitiligo (35), on the contrary, they may be associated with the onset or exacerbation of the condition (36, 37), which is consistent with the findings reported in our study.

We hypothesize that certain immunosuppressants may contribute to drug-induced vitiligo by modulating the IL-17 signaling pathway (31). Previous studies have shown that IL-17, produced by Th17, can directly antagonize factors essential for melanocyte function and survival, thereby potentially contributing to depigmentation (33). Secukinumab and ixekizumab, both IL-17A inhibitors, exert their therapeutic effects by blocking this pro-inflammatory cytokine and subsequently downregulating the Th17-mediated immune response (38). While this mechanism is beneficial in conditions like psoriasis—where Th17-driven inflammation is a key pathogenic factor—the inhibition of IL-17A may disrupt immune homeostasis (39). In certain contexts, such disruption could impair the balance between Th17 and Th1 responses, thereby breaking immune tolerance and promoting autoimmune reactions against melanocytes. This imbalance may ultimately trigger or exacerbate vitiligo (35). Indeed, cases of IL-17 antagonist-induced vitiligo underscore the delicate regulatory interplay between Th1 and Th17 pathways, where suppression of one effector arm may unintentionally potentiate the other (40).

Similarly, immunosuppressants targeting IL-23 may inadvertently promote melanocyte-targeted autoimmunity by modulating the Th17 pathway. IL-23 is a key cytokine responsible for the differentiation and stabilization of Th17 cells, playing a central role in immune-mediated inflammation (41, 42). Among these agents, ustekinumab (43)—a dual inhibitor of IL-12 and IL-23—along with guselkumab and risankizumab (44, 45), which specifically target the p19 subunit of IL-23, effectively suppress inflammatory responses by inhibiting Th17 cell activity and downstream cytokine production. These immunosuppressants have demonstrated significant clinical efficacy in treating Th17-driven diseases such as psoriasis (46). However, by selectively modulating the IL-23/Th17 axis, they may also disrupt peripheral immune tolerance, potentially leading to unintended autoimmune effects, including the development or exacerbation of vitiligo in susceptible individuals.

In the case of alemtuzumab, the mechanism appears distinct. Alemtuzumab is a monoclonal antibody that binds to the CD52 antigen, expressed on lymphocytes, and causes antibody-dependent cellular lysis, thereby depleting T and B lymphocytes (47). While this broad immune suppression is therapeutically beneficial in diseases such as multiple sclerosis (48), it may also lead to unintended consequences, including the development of autoimmune conditions such as vitiligo. One proposed mechanism is that the depletion and subsequent repopulation of immune cells disrupt immune homeostasis, thereby promoting the activation of autoreactive T cells. This hypothesis is supported by findings from Ruck T et al. (13), who observed that patients developing vitiligo following alemtuzumab treatment exhibited abnormally high proportions of activated and memory CD8^+^ T cells. These findings suggest a possible link between immune reconstitution and the emergence of autoimmunity. However, the precise immunopathological mechanisms underlying this phenomenon remain to be fully elucidated.

Our study reveals several demographic and clinical characteristics that may influence the observed association between immunosuppressant use and vitiligo. Firstly, among the 435 reports, a higher proportion of female patients compared to male patients was observed. Recent research indicates that there is no significant difference in prevalence between males and females (49). This phenomenon may be attributed to the fact that gender differences play a significant role in the immune system’s response. Research (50) indicates that women are more susceptible to autoimmune diseases compared to men, and this disparity is closely linked not only to sex hormones but also to genetic factors. Consequently, women are more likely to develop such diseases and may require long-term reliance on immunosuppressants to manage these conditions. Secondly, in terms of weight data, the majority of the information was not recorded, and the specific weight range distribution showed a small number of cases with underweight or overweight conditions. This result limits our analysis of the impact of weight on the relationship between immunosuppressants and vitiligo. Regarding age distribution, a higher proportion of patients aged between 18 and 65 years may be because this age group is more likely to be diagnosed with chronic diseases (51), thus requiring more medication and increasing the opportunity for reporting adverse drug events. The diversity in report sources, primarily physicians, may be due to their direct observation of AEs in clinical practice, as well as their responsibility and capability to report (52). Furthermore, in terms of geographical distribution, the United States accounts for 60.5% of the reports, likely due to its large population base, high rate of drug usage, and well-developed drug safety surveillance system (53). Next are other countries such as Canada, France, Germany, and Brazil, which may be related to the pharmaceutical market size, drug usage patterns, and regulatory environment in these countries. Lastly, in terms of indications, psoriasis, rheumatoid arthritis, and psoriatic arthropathy are the most commonly reported diseases, which may be associated with the immunosuppressants or immunomodulatory drugs commonly used by patients with these diseases (54), potentially contributing to an elevated reporting frequency of this AE.

Our study is subject to inherent limitations of pharmacovigilance databases. First, the retrospective, spontaneous reporting design precludes establishing definitive causality. Second, as the total number of exposed patients is unknown and under-reporting is inevitable, incidence rates cannot be calculated; thus, our results reflect reporting trends rather than absolute risk (55). Third, confounding factors, including concomitant medications and underlying comorbidities, cannot be fully adjusted for (55, 56). Finally, the substantial proportion of missing demographic data, including age (missing in 48% of reports), restricts the ability to perform comprehensive subgroup analyses. Despite these limitations, the consistency of the safety signal across multiple disproportionality analysis methods enhances the credibility of the observed associations.

In conclusion, our study highlights the importance of identifying potential adverse effects associated with immunosuppressants. The significant association between certain immunosuppressants—many of which impact key immune pathways, including the IL-17 signaling pathway and the IL-23/Th17 axis—and the onset of vitiligo warrants further investigation into the specific mechanisms involved (**Table 4**). This will not only deepen our understanding of these drug-induced adverse effects but also support the development of strategies to mitigate such potential safety issues. Hence, future studies should adopt prospective designs and gather more extensive data on drug exposure and the development of vitiligo to better validate and build upon our findings. Ultimately, our findings offer valuable insights to inform clinical decision-making and suggest future directions for research in this area.

**Table 4:** Medications investigated included in the present study and their mechanisms of action.

Medications investigated	Mechanism of action
L04A — IMMUNOSUPPRESSANTS	
L04AC — Interleukin inhibitors	
L04AC05 — Ustekinumab	Targets interleukins IL-12 and IL-23, inhibiting their activity to modulate immune responses and reduce inflammation.
L04AC10 — Secukinumab	Inhibits interleukin-17A (IL-17A), reducing inflammatory responses associated with autoimmune conditions.
L04AC13 — Ixekizumab	Binds to IL-17A cytokine, blocking its interaction with receptors and decreasing inflammation.
L04AC16 — Guselkumab	Selectively targets the p19 subunit of IL-23, inhibiting its signaling and reducing inflammatory processes.
L04AC18 — Risankizumab	Inhibits the IL-23 p19 subunit, leading to decreased cytokine production and diminished inflammation.
L04AG — Monoclonal antibodies	
L04AG06 — Alemtuzumab	Binds to CD52 antigen on lymphocytes, causing antibody-dependent cellular lysis and depletion of T and B cells.

## Data Availability

The original contributions presented in the study are included in the article/**Supplementary Material**. Further inquiries can be directed to the corresponding authors.

## Supplementary material

Supplementary Table 1 Integrated 2×2 Table, Formulas, and Thresholds for Four Analytical Methods.
